# Ductal Injection Does Not Increase the Islet Yield or Function after Cold Storage in a Vascular Perfusion Model

**DOI:** 10.1371/journal.pone.0042319

**Published:** 2012-08-10

**Authors:** Wataru Nakanishi, Takehiro Imura, Akiko Inagaki, Yasuhiro Nakamura, Satoshi Sekiguchi, Keisei Fujimori, Susumu Satomi, Masafumi Goto

**Affiliations:** 1 Division of Advanced Surgical Science and Technology, Tohoku University, Sendai, Japan; 2 New Industry Creation Hatchery Center, Tohoku University, Sendai, Japan; 3 Department of Pathology, Tohoku University School of Medicine, Sendai, Japan; University of Colorado, United States of America

## Abstract

Several studies have reported that pancreatic ductal preservation greatly improved the islet yield and function after cold storage. However, these studies were devoid of appropriate controls, such as vascular perfusion, which is routinely performed to preserve organs in the clinical setting. In this study, we created a vascular perfusion model using inbred rats, and investigated the effect of ductal injection on the islet yield and function after cold storage. Rat pancreases after 10 h cold ischemia were classified as follows: without ductal/vascular perfusion; with ductal injection; with vascular perfusion; and with ductal/vascular perfusion. The islet yield, function, viability, release of inflammatory mediators, and pathological changes in the exocrine tissues were assessed in the Hanks' Balanced Salt Solution (HBSS) model. The islet yield was also assesed by introducing University of Wisconsin Solution (UWS) and Histidine-Tryptophan-Ketoglutarate solution (HTK), which are the standard clinical preservation solutions. In the HBSS model, ductal injection and vascular perfusion significantly improved the islet yield compared with the control group. However, ductal injection showed no additional effects on the islet yield, function, viability and suppressing the release of inflammatory mediators when vascular perfusion was performed. Although ductal injection significantly decreased the apoptosis of exocrine cells, no beneficial effect on vacuolation was observed. In contrast, vascular perfusion significantly suppressed vacuolation in the exocrine tissues. Likewise, in the UWS and HTK model, ductal injection and vascular perfusion improved the islet yield compared with the control group. Nevertheless, the combination group showed no additional effects. These data suggest that ductal injection has no additional effect on islet yield and function after cold storage in a vascular perfusion model. We propose that ductal injection can be an effective and simple alternative for vascular perfusion prior to pancreas harvest, but is not necessary in most cases, since vascular perfusion is routinely performed.

## Introduction

Islet transplantation is a promising strategy for curing type 1 diabetic patients. Although Shapiro et al. greatly improved the clinical outcome [1], the current isolation procedures can recover less than 50% of the total islets within a pancreas. Therefore, two to four donors are generally needed to cure one diabetic patient. Furthermore, recent data indicate that the success rate of islet isolation is still less than 50%, even in the most advanced centers using refined techniques [2]. Hence, in order for islet transplantation to become a widespread therapy, better islet volume recovery is needed to achieve a higher success rate for diabetes reversal from one donor.

Although several factors may contribute to the failure of islet isolation, ischemic stress during pancreatic harvesting and preservation has been known to deteriorate islet recovery and function [3], [4], [5]. Therefore, a new method to alleviate ischemic stress is needed to obtain a sufficient islet mass from a single donor.

It has been reported that a pancreatic ductal preservation method greatly improved both the islet yield and function after cold ischemia [6], [7]. The authors of these reports speculated that one of the main advantages of ductal injection is an efficient distribution of collagenase due to better preservation of the pancreatic duct. Based on these data, ductal injection has already been used following the procurement of human pancreases in the clinical setting [2], [8], [9].

Of particular note, however, the previous studies on the effects of ductal injection were devoid of appropriate control groups, namely a vascular perfusion group. In the current clinical setting, vascular perfusion using cold preservation solutions is routinely performed to preserve organs at the time of harvest from cadaveric donors. It seems likely that vascular perfusion *per se* may have a beneficial influence on the islet yield and function. Therefore, to properly elucidate the effects of the ductal preservation method, experimental models with vascular perfusion need to be examined.

In the present study, we hypothesized that a ductal perfusion does not have an additional effect on protecting the pancreas from the ischemic stress when vascular perfusion is simultaneously performed. At the first step, we investigated the effects of vascular perfusion and/or ductal injection on the islet yield and function after a 10 h cold ischemia time (CIT) using the Hanks' Balanced Salt Solution (HBSS) in a vascular perfusion model of inbred rats, which were expected to show minimal individual differences. We also examined whether ductal injection could regulate the release of inflammatory mediators from the isolated islets. Futhermore in order to mimic clinical situation, University of Wisconsin Solution (UWS) and Histidine-Tryptophan-Ketoglutarate solution (HTK) were applied at the seconed step. In the UWS and HTK model, we assesed the effects of vascular perfusion and/or ductal injection on the islet yield after a 30 h and 10 h CIT.

## Results

### The effects of ductal injection and/or vascular perfusion on the yield of rat islets isolated under cold ischemic stress

**Figure 1:**
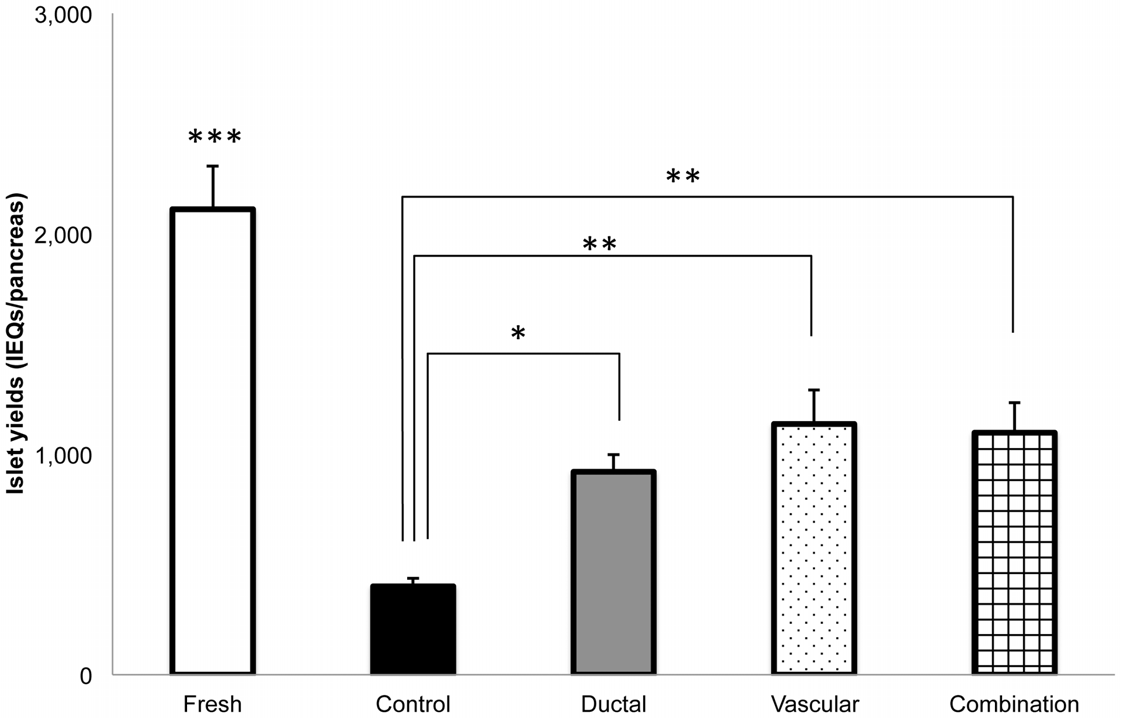
The effects of ductal injection and/or vascular perfusion on the islet yield (HBSS). In the HBSS model, the isolated islet yield was significantly increased in the ductal injection (gray bar: ductal group: n = 9), vascular perfusion (dotted bar: vascular group: n = 9), and ductal injection/vascular perfusion groups (squared bar: combination group: n = 10) compared with that of the cold ischemia control group (black bar: control group: n = 9). The islets isolated from pancreases with a negligible cold ischemia time (less than 10 min) served as the fresh control group (white bar: fresh group: n = 7). The islet yield of the fresh group was significantly higher than that of the other groups. No significant differences were observed among the ductal, vascular, and combination groups. *P<0.05, **P<0.005, ***P<0.0001.

**Figure 2:**
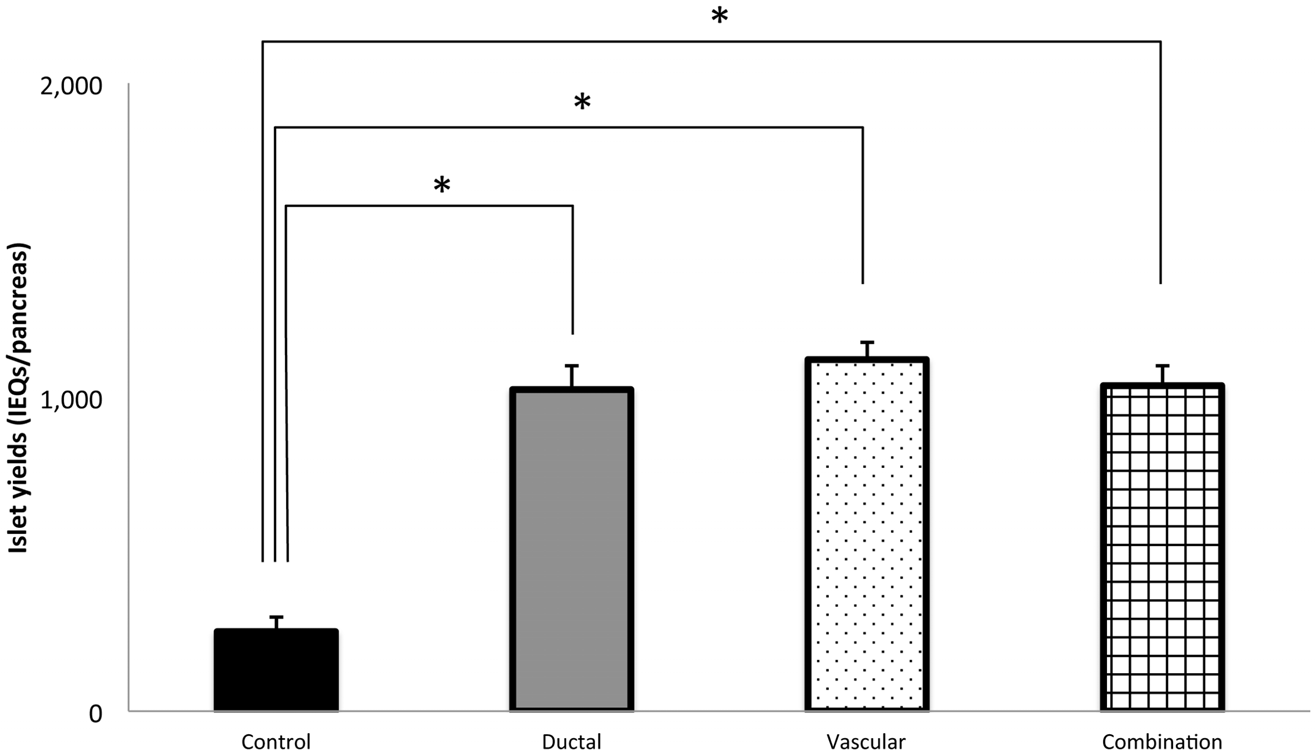
The effects of ductal injection and/or vascular perfusion on the islet yield (UWS). In the UWS model, the isolated islet yield significantly increased in the ductal injection (gray bar: ductal group: n = 10), vascular perfusion (dotted bar: vascular group: n = 9), and ductal injection/vascular perfusion groups (squared bar: combination group: n = 10) in comparison to that of the cold ischemia control group (black bar: control group: n = 10). No significant differences were observed among the ductal, vascular, and combination groups. *P<0.0001.

**Figure 3:**
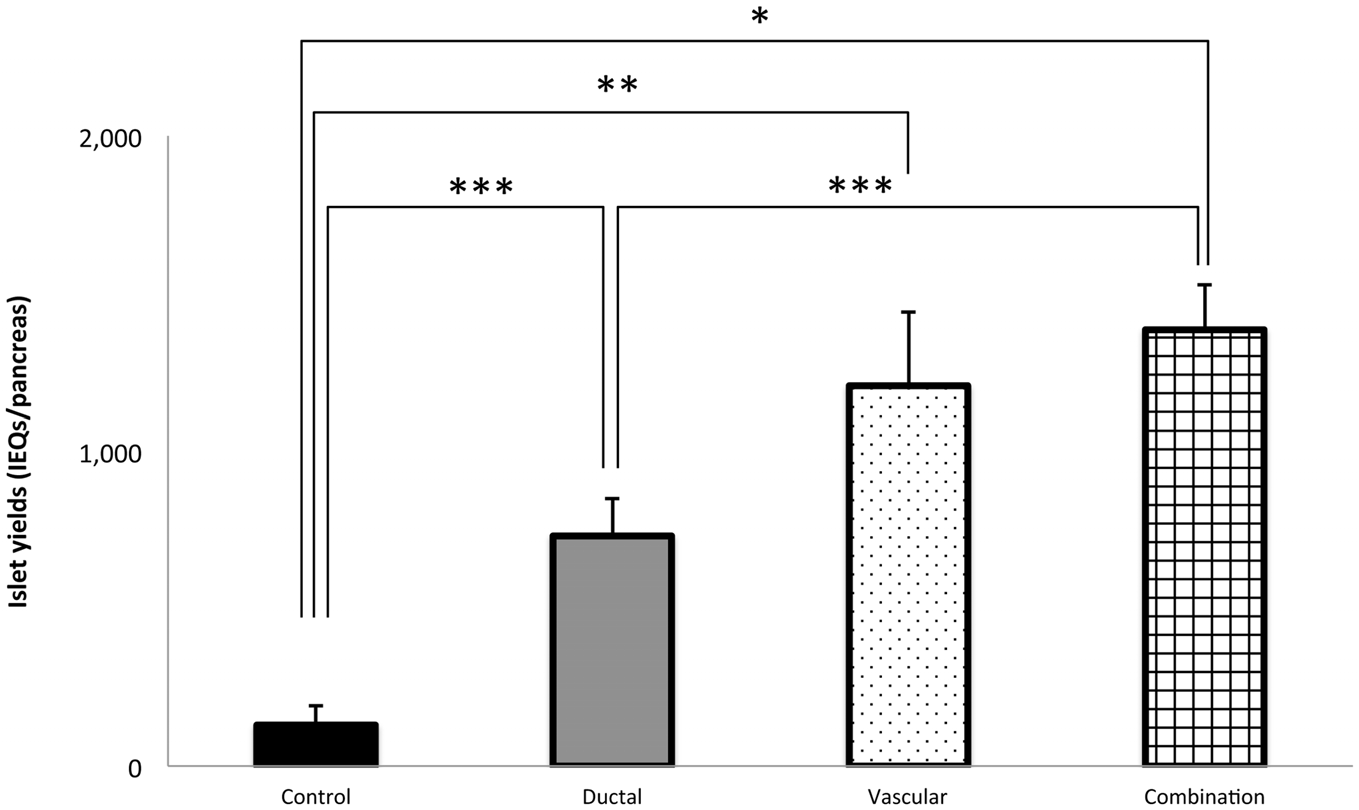
The effects of ductal injection and/or vascular perfusion on the islet yield (HTK). In the HTK model, the isolated islet yield significantly increased in the ductal injection (gray bar: ductal group: n = 7), vascular perfusion (dotted bar: vascular group: n = 7), and ductal injection/vascular perfusion groups (squared bar: combination group: n = 7) in comparison to that of the cold ischemia control group (black bar: control group: n = 7). Furthermore, the islet yield in both the vascular perfusion and ductal injection/vascular perfusion groups was significantly higher than that of ductal injection group. *P<0.0001, **P = 0.0002, ***P<0.05.

In the HBSS model (Fig. 1), the islet yield in the fresh isolation group was the highest (2,111±196 islet equivalents (IEQs), n = 7) among all the groups. In the ischemic groups, the islet yield was the lowest in the control group (400±38 IEQs, n = 9). Both ductal injection (920±78 IEQs, n = 9) and vascular perfusion (1,137±154 IEQs, n = 9) significantly improved the islet yield compared with the control group (P<0.05, P<0.005, respectively). The combination group (ductal injection+vascular perfusion; 1,097±137 IEQs, n = 10) also significantly improved the islet yield compared with the control group (P<0.005), but did not show any additional effect compared to the vascular perfusion group. In the UWS and HTK model (Fig. 2 and 3), the islet yield was the lowest in the control group (253±45 IEQs ; n = 10, 130±61 IEQs ; n = 7). Ductal injection (1,024±76 IEQs ; n = 10, 731±120 IEQs ; n = 7) and vascular perfusion (1,119±54 IEQs, n = 9, 1,206±234 IEQs ; n = 7) significantly improved the islet yield in comparison to the control group (P<0.0001, P<0.05, P<0.0001, P = 0.0002, respectively). The combination group (1,036±62, n = 10, 1,384±143 IEQs ; n = 7) also significantly improved the islet yield in comparison to the control group (P<0.0001), but did not show any additional effects compared to the vascular perfusion group.

### The effects of ductal injection and/or vascular perfusion on viability and function of rat islets isolated and exocrine tissues under cold ischemic stress

**Figure 4:**
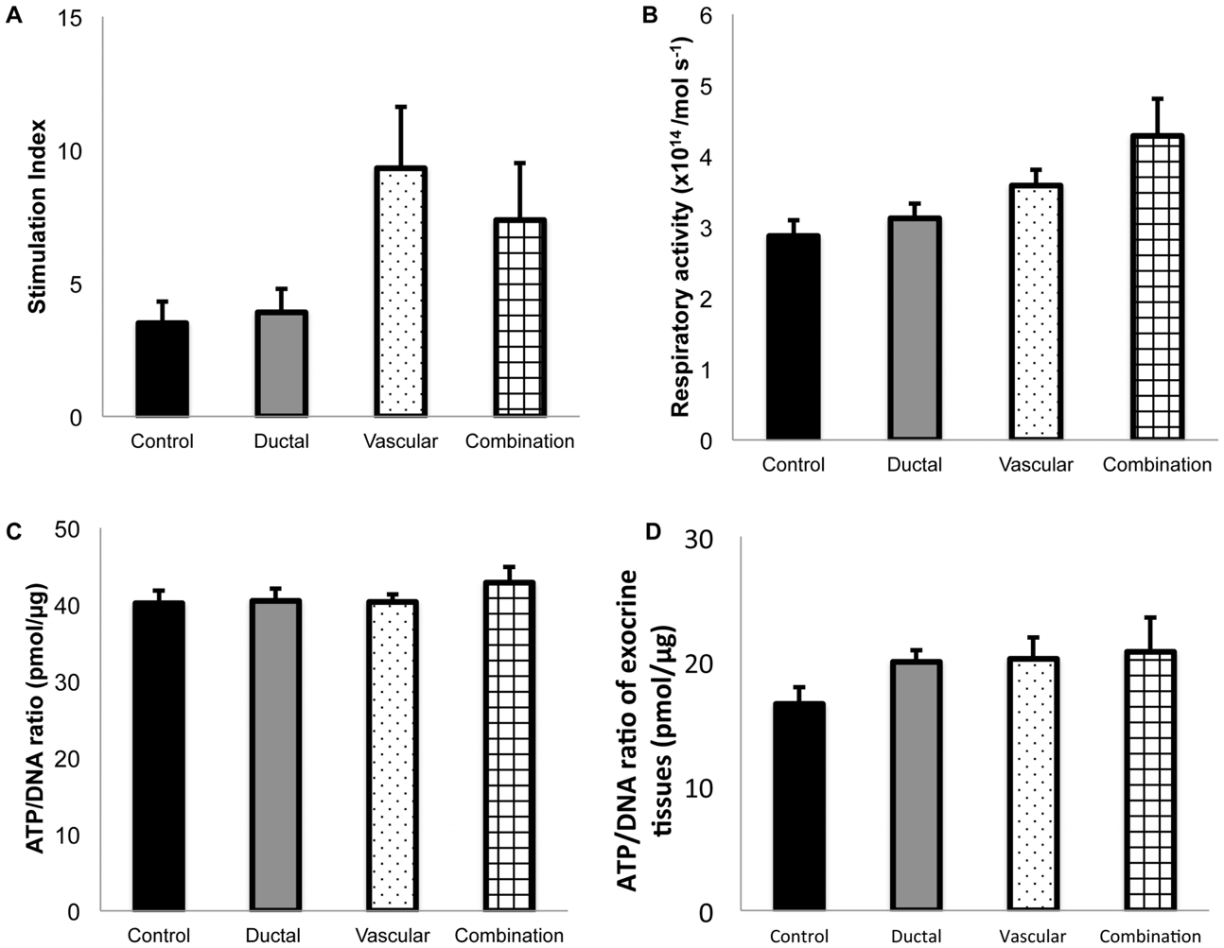
The effects of ductal injection and/or vascular perfusion on the function and viability of islets. A SGS test of islets isolated (A) was performed to evaluate the influence of cold ischemic stress on islet function. To examine the influence of cold ischemic stress on islet viability, the respiratory activity (B) and the ATP/DNA ratio (C) of isolated islets were measured. In all assays performed, no significant differences were detected among the groups. The ATP/DNA ratios of the exocrine tissues after cold preservation are shown (D). No significant differences were detected among the groups.

A static glucose stimulation (SGS) test (Fig. 4A) was performed to evaluate the influence of cold ischemic stress on islet function (stimulation index (SI): control 3.49±0.81 (n = 9), ductal injection 3.90±0.88 (n = 8), vascular perfusion 9.31±2.30 (n = 8), combination 7.36±2.14 (n = 7)). The SI in both the vascular perfusion and combination group was considerably higher than that of the other groups, but the differences among the 4 groups did not reach statistical significance (P = 0.051). To examine the influence of cold ischemic stress on islet viability, the respiratory activity (Fig. 4B) and the adenosine triphospahte (ATP)/deoxyribonucleic acid (DNA) ratio of isolated islets (Fig. 4C) were measured (respiratory activity: control 2.87±0.22×10^14^/mol s^−1^, ductal injection 3.12±0.20×10^14^/mol s^−1^, vascular perfusion 3.58±0.22×10^14^/mol s^−1^, combination 4.28±0.52×10^14^/mol s^−1^, n = 6, respectively ; ATP/DNA ratio: control 40.1±1.6 pmol/µg (n = 6), ductal injection 40.4±1.6 pmol/µg (n = 7), vascular perfusion 40.3±1.0 pmol/µg (n = 7), combination 42.8±2.0 pmol/µg (n = 6)). For all viability assays performed, no significant differences were detected among the groups.

To investigate the effects of ductal injection and/or vascular perfusion on the energy status of exocrine tissues, the ATP/DNA ratio of exocrine tissues was examined (Fig. 4D). The ATP/DNA ratios of exocrine tissues in all the perfusion groups (ductal injection: 19.9±1.0 pmol/µg (n = 7), vascular perfusion: 20.1±1.7 pmol/µg (n = 6), combination: 20.7±2.7 pmol/µg (n = 6)) were higher than that of the control group (16.5±1.4 pmol/µg (n = 7)), although no statistically significant differences were detected (P = 0.31).

### Immunohistochemical analyses

**Figure 5:**
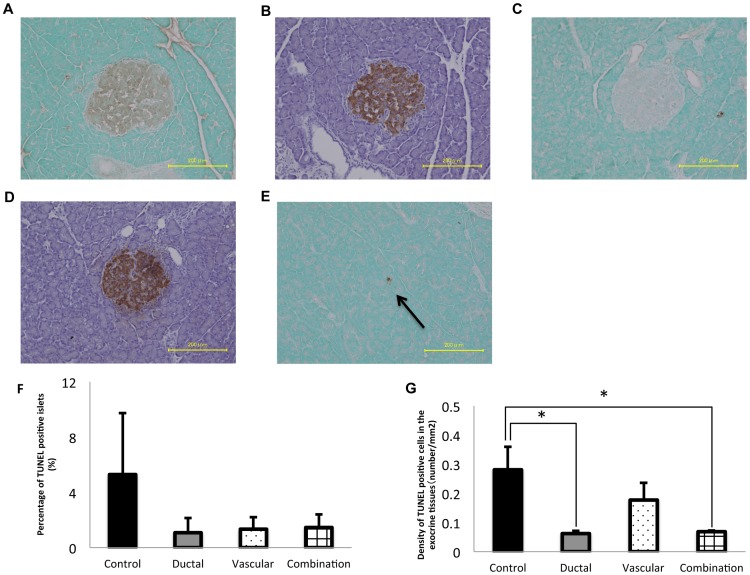
The TUNEL staining of the pancreatic islets and the exocrine tissues after cold storage. Representative of TUNEL positive islets (A), with insulin staining (B), and representative of TUNEL negative islets (C), with insulin staining (D) are shown. (E) The TUNEL positive cells in the exocrine tissues are shown (arrow). (F) The percentage of TUNEL positive islets (the number of TUNEL positive islets/the total number of islets *100) was calculated and evaluated. No significant differences were detected between groups. (G) The density of TUNEL positive cells in the exocrine tissues (the number of TUNEL positive cells/the total area of exocrine tissues in the sections) was calculated. The density of TUNEL positive cells in the ductal injection and combination groups was significantly lower than that of the control group. Magnification ×200. The data are shown as the means ± SE. *P<0.05.

**Figure 6:**
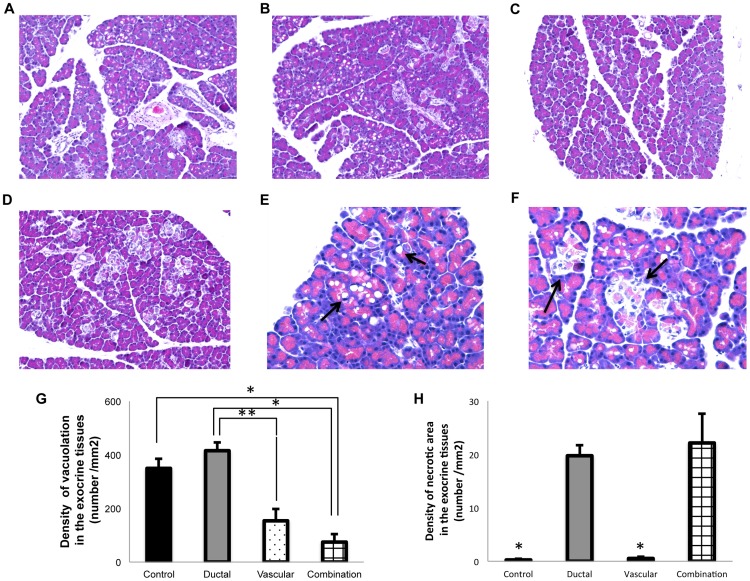
Evaluation of the vacuolation and necrotic area in the exocrine tissues. (A–D) Representative H&E images used to count the vacuolation and necrotic area in each group. Magnification ×200. (A) Control group, (B) Ductal injection group, (C) Vascular perfusion group, (D) Combination group. (E) H&E stained examples of vacuolation are shown (arrow). Magnification ×400. (F) H&E examples of necrotic areas in the exocrine tissues are shown (arrow). Magnification ×400. (G) The density of vacuolation in the exocrine tissues (number/mm^2^). The density of vacuolation in the exocrine tissues was significantly lower in the vascular and combination groups compared with the ductal group. The vacuolation density of the combination group was also significantly lower than that of the control group. The data show the means ± SE. *P<0.01 **P<0.05 (H) The density of the necrotic area in the exocrine tissues (number/mm^2^). The densities of the control and the vascular groups were significantly lower than that in the other groups. *P<0.01.

The DNA fragmentation of islets and exocrine tissues was evaluated by the TUNEL (TdT-mediated dUTP nick end labeling assay) to assess the extent of apoptosis. Representative examples are shown in Figs. 5A, C, and E. As summarized in Fig. 5F, the ratio of TUNEL positive islets tended to be higher in the control group, but the difference compared to the other groups did not reach statistical significance. On the other hand, the density of TUNEL positive exocrine cells in the ductal injection and combination groups was significantly lower than that of the control group, as shown in Fig. 5G (p<0.05). A pathological analysis revealed that the exocrine tissues showed vacuolation (Fig. 6E) and necrotic changes (Fig. 6F) after cold storage (Figs. 6A–D). Vacuolation in an organ after preservation can be considered a useful barometer reflecting the extent of damage due to ischemic stress [10]. As summarized in Fig. 6G, the density of vacuolation in the exocrine tissues was significantly lower in the vascular perfusion (154±45/mm^2^) and combination groups (74±31/mm^2^) compared with that of the ductal group (415±31/mm^2^) (P<0.05, P<0.01, respectively). The vacuolation density of the combination group was also significantly lower than that of the control group (349±35/mm^2^) (P<0.01). As summarized in Fig. 6H, necrotic changes were observed only in the ductal injection and combination groups, suggesting that these changes cannot be prevented by ductal injection alone.

### The effects of ductal injection and/or vascular perfusion on the release of inflammatory mediators from rat isolated islets under cold and warm ischemic stress

**Figure 7:**
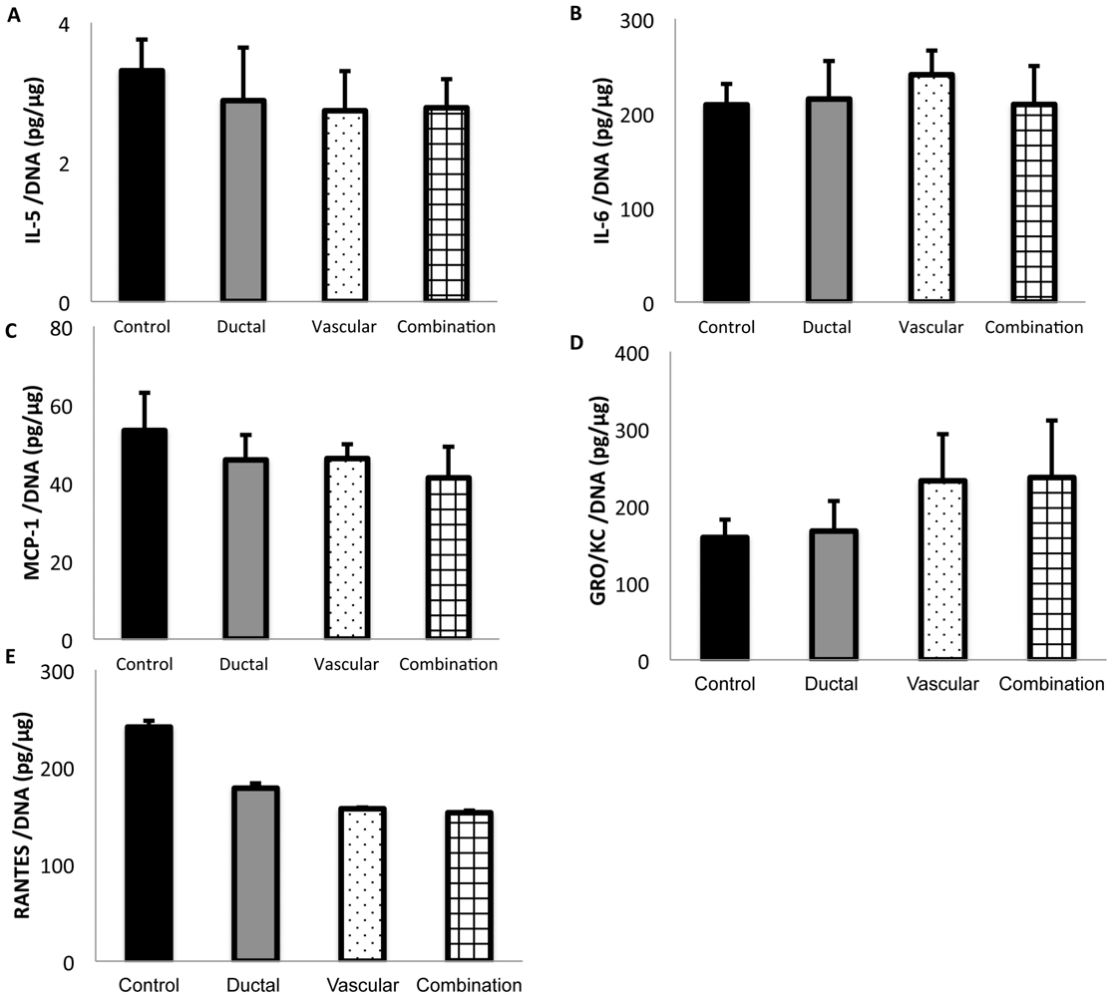
The effects of ductal injection and/or vascular perfusion on the release of inflammatory mediators. The inflammatory mediators released from 50 islets isolated from the pancreases subjected to 10 h cold ischemia and 30 min warm ischemia were cultured for 72 h and then examined (n = 8). IL-5 (A), IL-6 (B), MCP-1 (C), GRO/KC (D), and RANTES (E) could be detected in all samples. The data show the means ± SE. No significant differences were detected among the groups.

Isolated islets subjected to ischemic stress, especially warm ischemia [11], [12], are known to release several inflammatory mediators. Therefore, unlike other experiments, islets isolated from the pancreases subjected to 10 h cold ischemia and 30 min warm ischemia were used in this assay. As shown in Fig. 7, the expression levels of IL (interleukin) -5, IL-6, MCP-1 (monocyte chemotactic protein-1), GRO/KC (growth regulated protein/Keratinocyte derived chemokine), and RANTES (regulated upon activation, normal T cell expressed and secreted) could be detected and evaluated. No significant differences were detected among the various groups.

## Discussion

Several studies have reported that pancreatic ductal preservation greatly improved both the islet yield and function after cold ischemic storage [2], [6], [7], [9]. However, no studies have used a vascular perfusion model, which is the current clinical standard technique, to evaluate the significance of ductal injection. We hypothesized that one of the main mechanisms responsible for the beneficial effects of ductal injection might be the protection of the exocrine tissues from cold ischemic stress. However, it seemed likely that vascular perfusion would effectively protect the exocrine tissues from the cold ischemia as well. Therefore, in the present study, we applied a vascular perfusion model using inbred rats which are expected to show minimal individual differences, and found that ductal injection had no additional effect on either islet recovery or function at least in *in vitro* assays after cold storage when vascular perfusion was performed. Of particular note, the same tendency was observed when the potent preservation solution was applied.

Corroborating our hypothesis, the TUNEL staining in the present study revealed that cold ischemia substantially damaged the exocrine tissues, rather than the pancreatic islet cells. Moreover, it was also shown that ductal injection could significantly protect the exocrine tissues from the apoptotic changes. Unexpectedly, vascular perfusion had only a marginal effect on inhibiting the apoptotic changes of the exocrine tissues. However, vascular perfusion, unlike ductal injection, efficiently suppressed vacuolation in the exocrine tissues after cold preservation. In the present study, frequent vacuolation was observed in the pancreatic tissues after cold storage, while only a few apoptotic cells were detected. Considering the limited apoptotic changes, it may be speculated that vacuolation could be a useful marker to detect the early phase of ischemic damage. This finding is consistent with a previous report by Monbaliu et al [10]. They also found that substantial vacuolation in the hepatocytes was observed in the early phase of liver preservation.

In the present study, we also observed several necrotic areas in the pancreatic acini, but only in the ductal injection and combination groups. Given that no necrotic areas were detected in the control group, these changes seem to have been induced by the ductal injection. However, the previous studies reported that both pancreatic duct cells and the exocrine tissues were preserved when ductal injection was performed prior to pancreas preservation [6], [7], [8]. A possible explanation for this discrepancy is the solution used for ductal injection. Although it is still difficult to explain why no necrotic areas were detected in the vascular group in the present study, ductal injection using potent preservation solutions may prevent necrotic changes of the exocrine tissues if they are induced by the ductal injection.

Another possible explanation for the discrepancy between the present study and the previous studies is the animal model used. For islet isolation, collagenase injection through the pancreatic duct and distension of the pancreatic tissues are definitely critical and essential for pancreas digestion. In small animal models, collagenase delivery seems to be of less importance than in humans, even after cold storage. In large animals, it may have a stronger impact on the islet yield, since the ductal components and structures are complicated. Further investigations using both ductal and vascular perfusion models in a large animal setting are required to optimize the ductal injection procedure and to determine whether it can improve the islet viability or function.

In support of the previous findings [6], [7], the islet yield was significantly increased by introducing ductal injection. Nevertheless, the present study clearly showed that ductal injection, at least in this rat model, did not exert any additional effects on the islet yield or function when vascular perfusion was performed. However, these results simultaneously suggest that ductal injection has a similar protective effect on the pancreatic tissues, leading to protection from ischemic stress, similar to the protection provided by vascular perfusion. In other words, ductal injection can be a useful alternative when vascular perfusion is not possible or is not performed. In view of the clinical aspects of these methods, the ductal injection method may have advantages compared with vascular perfusion when anti-inflammatory agents are used for pancreas preservation, since it allows for an organ-specific approach.

It was previously reported that isolated islets subjected to cold ischemic stress produce inflammatory cytokines [13]. Duct cells are also known to produce inflammatory mediators, including tumor necrosis factor-alpha (TNF-a) [14]. The ductal injection method may prevent cytokine release from duct cells, therefore, we investigated the effects of ductal injection on the release of cytokines from the cultured islets subjected to cold ischemic stress. Although no beneficial effect was observed in this study, the use of a potent preservation solution may lead to a different outcome. Therefore, this issue is a topic of interest for future studies.

In conclusion, the present data suggest that ductal injection has no additional effect on the islet yield and function after cold storage when vascular perfusion is performed in rat model. However, we propose that ductal injection can be used as an effective and simple alternative for vascular perfusion prior to pancreas harvesting. Further investigations using both ductal and vascular perfusion models in a large animal setting are required.

## Materials and Methods

### Animals

All animals in this study were handled in accordance with the Guide for the Care and Use of Laboratory Animals published by the National Instituted of Health [15]. The animal studies were approved by the Animal Care and Use Committee of the Tohoku University (approved protocol ID: 2011 NICHe-Animal-2). Islets were isolated from inbred male Lewis rats (Japan SLC Inc., Shizuoka, Japan) weighing 268 to 304 g.

### Pancreas procurement, perfusion, and preservation

In the present study, HBSS, UWS (Bristol-Myers Squibb Pharmaceutical Limited, Dublin, Ireland), and HTK (Dr. Franz Kohler Chemie GmbH, Bensheim, Germany) were used for vascular perfusion, ductal injection, and pancreas preservation. HBSS, which is a simple buffer solution, was employed to focus on the comparison between the ductal injection and vascular perfusion procedures *per se*. UWS and HTK are potent preservation solutions and have commonly been used for clinical islet transplantation. The donor rats were anesthetized using isoflurane (Abbott Japan Co., Ltd., Tokyo, Japan) to minimize suffering. After injection of 100 IU of heparin, the common bile duct was cannulated using a 24 G SURFLO I.V. catheter (TERUMO, Tokyo, Japan) and fixed with surgical silk. The pancreas was perfused via cannulation of the abdominal aorta using a 24 G SURFLO I.V. (TERUMO, Tokyo, Japan) and then it was flushed with 50 ml of cold HBSS at a pressure of 100 cm H_2_0 or cold UWS at 5 ml/min using a syringe pump (TERUMO, Tokyo, Japan) after clamping the abdominal aorta above the celiac axis. Immediately after starting perfusion, the intrathoracic inferior vena cava (IVC) was dissected for outflow. At the aorta clamp, crushed ice was placed into the abdominal space to reduce the organ temperature during perfusion. In the groups without vascular perfusion, crushed ice was placed similarly, and the rat was left for 7 min in the HBSS model or 10 min in the UWS and HTK model (the same duration as the perfusion time). After vascular perfusion, the pancreas, along with the spleen, duodenum and stomach, was removed as *en bloc*, and ductal injection was performed via slow injection of 0.5 ml of HBSS, UWS, or HTK through a common bile duct catheter. The distal bile duct was not clamped during a ductal perfusion to avoid excess pressure. And also, the catheter remained open during the entire preservation period. The pancreas was preserved in HBSS, UWS, or HTK at 4°C. In the present study, the CIT was defined as the time from clamping the aorta to the initiation of islet isolation. Pancreases were classified into 5 groups. In the fresh group, the pancreases were immediately isolated. The pancreases subjected to 10 h CIT in the HBSS and HTK model or 30 h CIT in the UWS model were classified as follows: with neither ductal injection nor vascular perfusion (control group); with ductal injection (ductal group); with vascular perfusion (vascular group); with ductal injection and vascular perfusion (combination group). In the warm ischemia time (WIT) model, the crushed ice was removed at the end of vascular perfusion, and the rat was left for 30 min. After that, the pancreas was procured similarly to the CIT model and preserved for 10 h in 4°C HBSS.

### Islet isolation and counting

The pancreas was distended using 1 mg/ml of a collagenase (Sigma type V; Sigma Chemicals, St Louis, MO, USA) solution in 10 ml HBSS through a common bile duct catheter. After the spleen, duodenum and stomach were removed, the pancreas was digested (12 min, 37°C). Thereafter, density gradient centrifugation was performed as previously reported [16]. The IEQs were counted under a scaled microscope using diphenylthiocarbazone (Wako, Osaka, Japan). One IEQ was the islet mass equivalent to a spherical islet of 150 µm in diameter.

### In vitro evaluation of islets and exocrine tissues

A SGS test was performed on the isolated islets as an *in vitro* test for islet function, as descrived previously [17]. The SI was defined as the ratio of the total amount of insulin secreted during the high glucose (16.7 mM) stimulation and that released during low glucose stimulation (1.67 mmol/l). Insulin was mesured by using a Rat Insulin ELISA kit (Mercodia AB, Uppsala, Sweden).

The ATP/DNA ratios of 30–40 islets in each group and of the exocrine tissues were measured after cold preservation. Forty exocrine tissue blocks (200 µm in diameter) were selected for each assay. The ApoGlow™ kit (Lonza Rock-land Inc., Rockland, ME, USA) was used for the ATP measurements, as described previously [18]. Using the same sample, the DNA content was measured using the DNA Quantify kit (Primary cell, Sapporo, Japan) as previously reported [16]. For the respiratory assay, scanning electrochemical microscopy, which automatically measured the reduced current of far and near points of the samples based upon the spherical diffusion theory, was used as previously reported [18]. Briefly, the respiratory activity of 5 islets in each group was calculated by evaluating the differences in the reduction current around the samples using 2–4 µm platinum-coated microelectrodes.

### Cytokine levels in the supernatant of cultured islets isolated from the pancreases subjected to ischemic stress

After isolation with cold and warm ischemia, 50 islets were collected from each ischemic group and placed into 48 well dishes (Becton Dickinson, Mountain View, CA, USA) with 250 µl of RPMI 1640 containing 5.5 mmol/L glucose and 10% fetal bovine serum, at 37°C in CO_2_ and humidified air for 72 h. After incubation, the supernatants were collected and frozen at −80°C until use. The islets in the wells were collected to measure the DNA content as previously described [16]. Cytokine levels were assessed by using the Milliplex® MAP Kit Rat Cytokine/Chemokine Magnetic Bead (Millipore, MA, USA) and Bio-plex200 (Bio-Rad Laboratories Inc., CA, USA).

### Imuunohistochemical analyses

Pancreases from the ischemic groups were placed into 4% paraformaldehyde and embedded in paraffin wax. DNA fragmentation *in situ* was assessed by the Apoptosis Detection Kit (TREVIGEN Inc., MD, USA). Slides (six sections per pancreas and three pancreases per group) were subjected to the TUNEL assay, and evaluated with regard to islets and exocrine tissues. In each section, the percentage of positive islets (the number of TUNEL-positive islets/the total number of islets) and the density of positive cells in the exocrine tissues (the number of TUNEL-positive cells counted in each section/the area of each section calculated by a digital microscope (BZ-9000, KEYENCE, Japan)) were determined. The vacuolation and the necrotic area in the exocrine tissues were also examined. Slides stained with hematoxylin and eosin (H&E) (two sections per pancreas and three pancreases per group) were examined. The density of vacuolation (the number of vacuoles in the exocrine tissues/the area of exocrine tissus in each field calculated by a digital microscope (BZ-9000, KEYENCE, Japan)) and the necrotic area (the number of necrotic cells/the area of exocrine tissues in each field calculated by a digital microscope (BZ-9000, KEYENCE, Japan)) in 10 random fields of each sample were calculated at 200-fold magnification.

### Statistical analysis

All results are reported as the means ± standard error (SE). A one-way ANOVA followed by the Tukey test was used for the statistical evaluations. A *P* value<0.05 was considered to be significant.
